# Confined placental mosaicism: implications for fetal chromosomal analysis using microarray comparative genomic hybridization

**DOI:** 10.1002/pd.4255

**Published:** 2013-11-14

**Authors:** Evangelia Karampetsou, Deborah Morrogh, Terry Ballard, Jonathan J Waters, Nicholas Lench, Lyn S Chitty

**Affiliations:** 1 NE Thames Regional Genetics Service, Great Ormond Street Hospital for Children NHS Foundation Trust, LondonUK; 2 TDL Genetics, The Doctors LaboratoryLondon, UK; 3 UCL Institute of Child HealthLondon, UK; 4 University College Hospital NHS Foundation TrustLondon, UK; 5 Great Ormond Street Hospital for Children NHS Foundation TrustLondon, UK

The utility of array comparative genomic hybridization (CGH) testing in prenatal diagnosis has been recently described^1^,^2^ with potential advantages, including improved detection of pathogenic chromosomal rearrangements following rapid analysis of uncultured chorionic villi or amniocytes. Whilst some of the technical difficulties encountered in the prenatal setting, such as DNA extraction for rapid results and interpretation of calls, have already been discussed, others, such as confined placental mosaicism (CPM) are yet to be evaluated.

Confined placental mosaicism for aneuploidies, and whole chromosome arm rearrangements, is a well-established phenomenon in chorionic villus samples (CVS), with a reported incidence of 1–2%.^3^ However, so far as we are aware, CPM for submicroscopic changes found by microarray testing has not been previously reported. Here, we report a case of CPM involving a deletion of exons 7–10 of the *STS* gene detected following analysis of array CGH results obtained from uncultured chorionic villi. The mosaic deletion was confirmed by an independent method [Multiplex Ligation-dependent Probe Amplification (MLPA)]. The *STS* deletion was not subsequently detected by analysis of cultured chorionic villus cells (CVS-CC) or neonatal blood obtained after delivery at term, confirming CPM.

Chorionic villus sampling was performed following sonographic detection at 12-week gestation of an increased nuchal translucency of 4.2 mm, absent nasal bone, fixed flexed knees and abnormal lower extremities. The CVS was carefully prepared by microscopic dissection, and the villi were then finely macerated and mixed. The macerated villi were used to establish cell cultures for karyotyping and to extract DNA for quantitative fluorescent polymerase chain reaction (QF-PCR) aneuploidy testing (glass bead extraction method^4^ and in-house developed QF-PCR method^5^ as described previously) and for microarray analysis [extraction using the QIAcube instrument (Qiagen, Germany)]. Parental blood samples were also obtained, and DNA was extracted using the FLEX STAR instrument (AutoGen, USA). After completion of karyotyping, cells were further cultured, and DNA was extracted using the iGENatal extraction kit (iGEN, Spain) and stored. Neonatal blood was obtained, and DNA was extracted using the QIAsymphony instrument (QIAGEN, Germany). Microarray testing was performed on DNA from direct CVS, CVS-CC, neonatal blood, maternal blood and paternal blood. All microarray testing was performed using sex-matched control DNA and the NimbleGen CGH 12 × 135 K WGT v3.0 whole genome array platform at a resolution of approximately 0.2 Mb in the HG19 build. Results were confirmed using the SALSA MLPA P160 STS kit (MRC-Holland, the Netherlands), which contains probes for each exon (1–10) of the *STS* gene, as well as other control probes on the X chromosome. MLPA data were analysed using the GeneMarker v1.91 software and intra-sample normalisation was performed.

The QF-PCR result was consistent with disomy 13, 18 and 21 and a male (XY) fetus. The karyotype result was normal male, 46, XY. The direct CVS microarray result indicated a 332 kb loss for the short arm of chromosome X with breakpoints within Xp22.31 [arr Xp22.31(7,223,740–7,555,450)×0] (log2 ratio: −1.52). This loss disrupted the *STS* (steroid sulphatase, OMIM #300747) gene, indicating a deletion of exons 8, 9 and 10, as well as possible deletion of exon 7 (Figure 1A). This deletion predicts a likely *STS* deficiency syndrome (X-linked ichthyosis, OMIM #308100) phenotype in a male fetus.^6^ Parental follow-up using microarray indicated no evidence of copy number change in this region in either parent, suggesting the *de novo* origin of this copy number loss in the fetus (Figure 1A). In view of the likely pathogenic nature of this deletion, the result was reported following discussion with our clinical review panel. Given the small size of the loss on chromosome X, there were no BAC (bacterial artificial chromosome) clones available within the deleted region, and therefore, confirmatory FISH (fluorescence *in situ* hybridization) testing was not performed.

**Figure 1:**
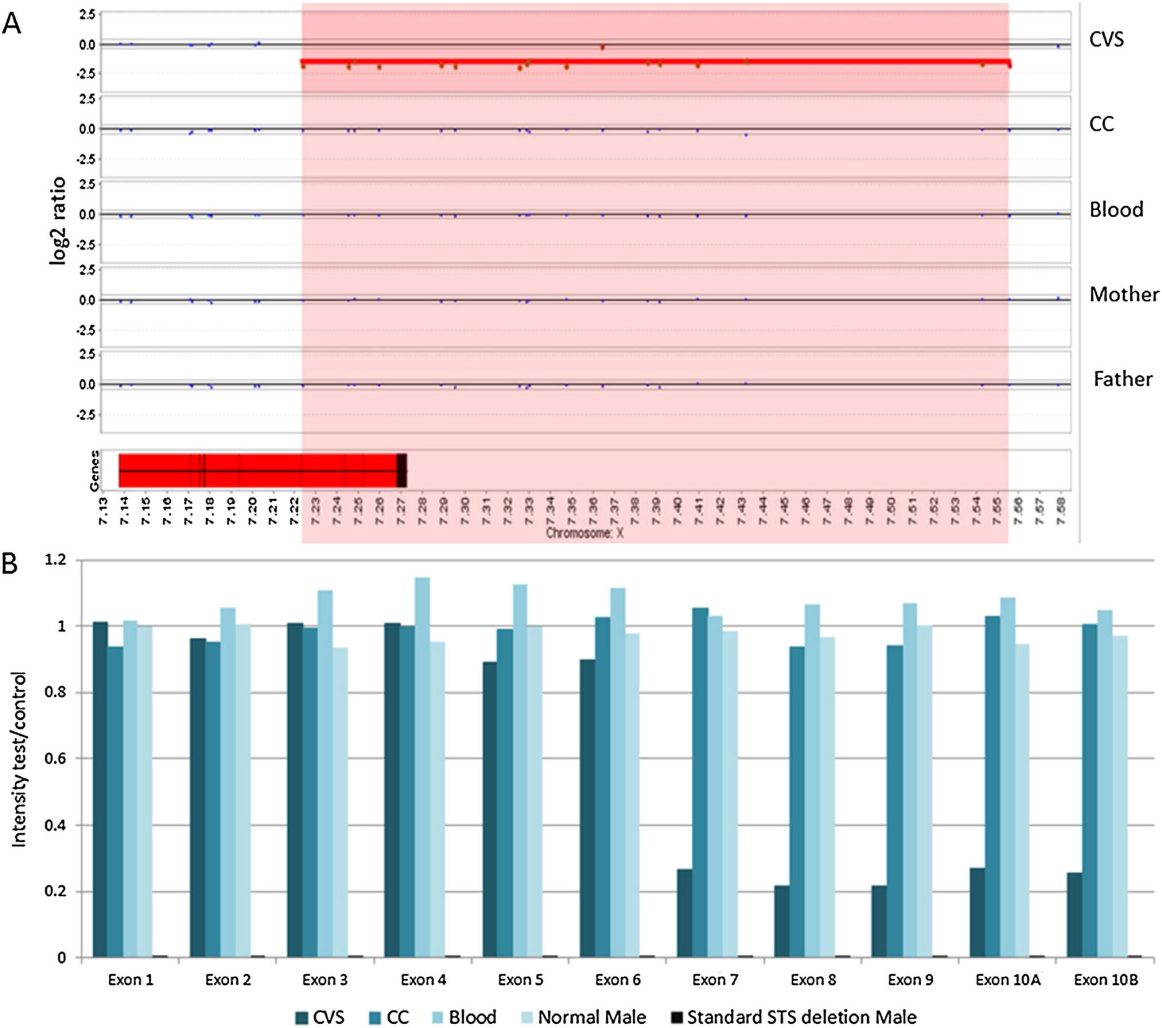
(A) Array CGH results. Magnification of the *STS* gene and copy number loss on the X chromosome at Xp22.31. The deletion region is highlighted in red. The *STS* gene is shown as a red block at the bottom left. Exons are shown as black lines within the *STS* gene box. A copy number loss disrupting the *STS* gene is identified in the direct chorionic villus sample (CVS). This loss included exons 8, 9 and 10 of the *STS* gene. Exon 7 is located adjacent to the call, but before the next normal probe, thus it could also be deleted. The results from DNA from CVS-CC (CC), neonatal (Blood), maternal (Mother) and paternal (Father) blood samples indicate no evidence of this copy number loss. Therefore, the deletion is *de novo* and confined to the placenta. (B) Multiplex Ligation-dependent Probe Amplification results for the *STS* locus. Only proband direct CVS (CVS), CVS-CC (CC) and neonatal blood (Blood) are shown here and are compared with a normal male sample and a sample with a known standard *STS* deletion. An intensity ratio between 0.8 and 1.2 corresponds to a normal copy number (i.e. 1 copy in males). Males carrying the deletion will show no amplification and an intensity ratio of 0 (0 copies). It is clear that exons 7–10 of the *STS* gene are deleted in a mosaic state in the direct CVS sample

The pregnancy continued and at birth the neonate had no sign of ichthyosis and subsequent microarray testing on DNA from neonatal blood failed to confirm the deletion disrupting the *STS* locus. Stored DNA from CVS-CC was also tested by microarray, and there was again no evidence of the deletion (Figure 1A). All array results met internal quality control standards.

The array results from the direct CVS, CVS-CC and neonatal blood all share the same eight regions of copy number variation, as well as two rare variants of unknown significance, both of which were maternally inherited, thereby eliminating the possibility of a sample mix-up.

Because the detection of mosaicism by array CGH can be problematic,^7^ we compared our results in this patient with positive controls from our own laboratory database. Four previous unrelated male probands with validated *STS* copy number losses detected postnatally were examined and showed an average log2 ratio of −2.5 (ranging from −2.25 to −2.8). The log2 ratio of the atypical partial *STS* deletion in the direct CVS presented in this report was −1.52, indicating that the copy number loss was mosaic in the direct CVS preparation, consistent with the admixture of two distinct cell lineage populations. MLPA analysis of the direct CVS, CVS-CC, neonatal blood and parental blood DNA, using the same direct approach, confirmed that the *STS* copy number loss seen by array CGH was not a technical artefact, involved exons 7–10 and was confined to the placenta in a mosaic state (Figure 1B).

To our knowledge, this is the first report of a potentially clinically significant copy number loss confined to the placenta and limited to the cytotrophoblast cell lineage detected by array CGH and subsequently confirmed by MLPA in a prenatal setting. DNA extracted from direct CVS contains a mixture of two cell populations derived from the cytotrophoblast and mesenchymal core. Mesenchymal cells, that grow in culture and are more likely to be representative of the fetal genotype, are estimated to contribute up to 40–50% to the DNA extracted from mid-trimester intermediate villi.^8^ Our microarray results suggest that the microdeletion on chromosome X arose during placentation and was confined to the cytotrophoblast.

Microarrays have only recently been introduced into clinical service for prenatal diagnosis, and so the frequency of discrepant results obtained by analysis of uncultured CVS and other sources of genetic material are as yet unknown. Our report highlights the possibility of CPM for submicroscopic rearrangements and suggests that precautions should be taken to minimise this risk. We would suggest that this case demonstrates the need for DNA extraction from a larger portion of the whole villi to maximise representation of the whole sample and to minimise discrepant results. Previous experience suggests that particular care is required to ensure that the mesenchymal core DNA fraction is sufficiently well represented to avoid such results.^9^ Although this protocol was followed in the case reported here, we still encountered discrepant results. Therefore, microarray results obtained from DNA extracted from uncultured CVS in the absence of clearly identified clinical findings (e.g. relevant prenatal sonographic findings) should be interpreted with caution. We suggest that, in the absence of such supporting evidence, follow-up and confirmation of potentially clinically significant copy number changes identified in DNA obtained from uncultured CVS should be performed on CVS-CC or amniotic fluid prior to reporting. In cases where CPM is suspected on microarray, FISH would be the preferred method for confirmation, as long as cultured cells and appropriate FISH probes are available. In addition, given the selective growth of the mesenchymal core cells in culture, the use of CVS-CC as the preferred tissue for microarray testing would minimise the incidence of CPM and false-positive findings. However, in a clinical diagnostic service, the use of CVS-CC instead of CVS would result in a delay in reporting of the urgent prenatal results. Moreover, cultural artefacts may arise that would not represent the true fetal genotype.^10^ The incidence of such artefacts would increase with prolonged culturing. In diagnostic laboratories, good practice would be to include in reports a standard rider stating that CPM cannot be excluded.

**WHAT'S ALREADY KNOWN ABOUT THIS TOPIC?**

- Use of microarray for prenatal diagnosis increases the detection of pathogenic chromosomal rearrangements.
- CPM is a well-established phenomenon occurring in 1–2% of CVS.

**WHAT DOES THIS STUDY ADD?**

- CPM can occur for submicroscopic copy number changes detected by array CGH in DNA from uncultured CVS. In order to minimise the risk of false-positive results in these cases, detected copy number changes should be confirmed in a different source of material, such as cultured cells.

## References

[b1] Shaffer LG, Dabell MP, Fisher AJ (2012). Experience with microarray-based comparative genomic hybridization for prenatal diagnosis in over 5000 pregnancies. Prenat Diagn.

[b2] Wapner RJ, Lese Martin C, Levy B (2012). Chromosomal microarray versus karyotyping for prenatal diagnosis. N Engl J Med.

[b3] Hahnemann JM, Vejerslev LO (1997). European collaborative research on mosaicism in CVS (EUCROMIC) – Fetal and extrafetal cell lineages in 192 gestations with CVS mosaicism involving single autosomal trisomy. Am J Med Genet.

[b4] Holgado E, Holgado B, Liddle S (2009). A novel method for extracting DNA from chorionic villus samples for use in CVS-PCR, which ensures complete villus dissociation. Prenat Diagn.

[b5] Levett LJ, Liddle S, Meredith R (2001). A large-scale evaluation of amnio-PCR for the rapid prenatal diagnosis of fetal trisomy. Ultrasound Obstet Gynecol.

[b6] Hernandez-Martin A, Gonzalez-Sarmiento R, De Unamuno P (1999). X-linked ichthyosis: an update. Br J Dermatol.

[b7] Miller DT, Adam MP, Aradhya S (2010). Consensus statement: chromosomal microarray is a first-tier clinical diagnostic test for individuals with developmental disabilities or congenital anomalies. Am J Med Genet.

[b8] Mann K, Kabba M, Donaghue C (2007). Analysis of a chromosomally mosaic placenta to assess the cell populations in dissociated chorionic villi: implications for QF-PCR aneuploidy testing. Prenat Diagn.

[b9] Waters JJ, Mann K, Grimsley L (2007). Complete discrepancy between QF-PCR analysis of uncultured villi and karyotyping of cultured cells in the prenatal diagnosis of trisomy 21 in three CVS. Prenat Diagn.

[b10] Yatsenko SA, Davis S, Hendrix NW (2013). Application of chromosomal microarray in the evaluation of abnormal prenatal findings. Clin Genet.

